# Mechanical Metamaterials for Handwritten Digits Recognition

**DOI:** 10.1002/advs.202308137

**Published:** 2023-12-25

**Authors:** Lingling Wu, Yuyang Lu, Penghui Li, Yong Wang, Jiacheng Xue, Xiaoyong Tian, Shenhao Ge, Xiaowen Li, Zirui Zhai, Junqiang Lu, Xiaoli Lu, Dichen Li, Hanqing Jiang

**Affiliations:** ^1^ State Key Laboratory for Manufacturing Systems Engineering Xi'an Jiaotong University Xi'an 710049 China; ^2^ School of Engineering Westlake University Hangzhou Zhejiang 310030 China; ^3^ Westlake Institute for Advanced Study Hangzhou Zhejiang 310024 China; ^4^ School of Aeronautics and Astronautics Zhejiang University Hangzhou Zhejiang 310027 China; ^5^ School for Engineering of Matter Transport and Energy Arizona State University Tempe Arizona 85287 USA; ^6^ Department of Physics Shaoxing University Shaoxing 312000 China; ^7^ Department of Physics Zhejiang Normal University Jinhua 321000 China; ^8^ Research Center for Industries of the Future Westlake University Hangzhou Zhejiang 310030 China

**Keywords:** 3D printing, image recognition, kirigami, mechanical metamaterial, non‐electrical

## Abstract

The increasing needs for new types of computing lie in the requirements in harsh environments. In this study, the successful development of a non‐electrical neural network is presented that functions based on mechanical computing. By overcoming the challenges of low mechanical signal transmission efficiency and intricate layout design methodologies, a mechanical neural network based on bistable kirigami‐based mechanical metamaterials have designed. In preliminary tests, the system exhibits high reliability in recognizing handwritten digits and proves operable in low‐temperature environments. This work paves the way for a new, alternative computing system with broad applications in areas where electricity is not accessible. By integrating with the traditional electronic computers, the present system lays the foundation for a more diversified form of computing.

## Graphical abstract

A non‐electrical mechanical metamaterial to achieve a neural network empowering handwritten number recognition without an electricity supply is presented. By overcoming the challenges of mechanical signal transmission efficiency and intricate layout design, a mechanical neural network based on kirigami metamaterial is achieved. This work provides an effective method for various science and engineering applications where electricity is not accessible.

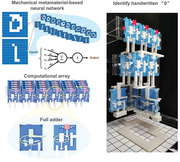

## 1 Introduction

The essence of intelligence is computing, which can take various forms, including electronic,^[^ ^1^ ^]^ mechanical,^[^ ^2^ ^]^ optical,^[^ ^3^, ^4^ ^]^ biological,^[^ ^5^ ^]^ pneumatic,^[^ ^6^ ^]^ fluidic,^[^ ^7^ ^]^ and many more forms. Currently, we are witnessing a glorious era of electronic‐based artificial intelligence (e.g., ChatGPT),^[^ ^8^ ^]^ where electrons become the information carriers, and electricity is the energy source that processes the signals. Similarly, in optical computing, photonic information is handled and carried by means of light quantum.^[^ ^9^ ^]^ Within this context, mechanical computing has always been a modest candidate. While a precursor to what constitutes modern artificial intelligence today, pure mechanical computing is presently finding new uses, particularly under conditions where electrical or optical computing are non‐starters. Mechanical computing is reliable in a range of scenarios, including but not limited to, harsh circumstances or extra‐terrestrial planets where electricity is beyond reach and/or in extreme environmental conditions where factors such as moisture, electromagnetic interference, radiation, and volatile temperatures hinder electricity‐based operations.

To date, mechanical computing, however, has received relatively little attention compared to mainstream electrical computing systems. This is partly because of the absence of a basic operational methodology that can successfully demonstrate its full benefits and its narrow focus on piecemeal components that constitute the basics of mechanical computing. These components include memory storage,^[^ ^10^ ^]^ logic gates, mechanical integrated circuit^[^ ^11^ ^]^ and common calculation modules^[^ ^6^, ^12^, ^13^, ^14^, ^15^ ^]^ based on soft components,^[^ ^6^ ^]^ origami,^[^ ^16^, ^17^ ^]^ and bar links.^[^ ^18^, ^19^, ^20^ ^]^ At present, two main obstacles hinder mechanical computing development. The first is the prevailing belief that a bottom‐up design starting from logic calculations and then assembling the whole, akin to electrical systems, is the only approach to developing a reliable system. The second barrier relates to the low transmission efficiency of mechanical signals^[^ ^21^ ^]^ compared to electrical signals, which are faster and more reliable. This study presents a new type of signal‐amplifying mechanism and a concise space topology of mechanical computing–all of which have remained unexplored.

In this paper, we demonstrate a novel mechanical neural network based on 3D printed bistable kirigami metamaterials to achieve a non‐electricity‐based artificial intelligence system without bulky assembled machinery usually composed of spring and rotary components. The developed mechanical computing empowers the coding of a simple handwritten number recognition exercise through specific neural network algorithms and the corresponding hardware assembly, all without electricity. A top‐down design methodology is used to simplify the structure of the mechanical modules, which stands in contrast to previously reported works^[^ ^22^, ^23^, ^24^ ^]^ that mostly rely on a bottom‐up design from basic logic gates. A concise topological layout is used to construct the clusters of these non‐electrical modules to achieve a mechanical neural network. To enhance the transmission efficiency of mechanical signals, the modules rely on unique 3D‐printed kirigami‐based mechanical metamaterials for amplifying both input displacement and force. This study represents a milestone in mechanical artificial system development because of its top‐down methodology and potential application in environments that cannot support electricity‐based operations for artificial intelligence. It provides an effective method for constructing more powerful and versatile computing forms with richer capacities.

## 2 Results

### 2.1 Overview of Constructed Non‐Electrical Neural Network

**Figure 1:**
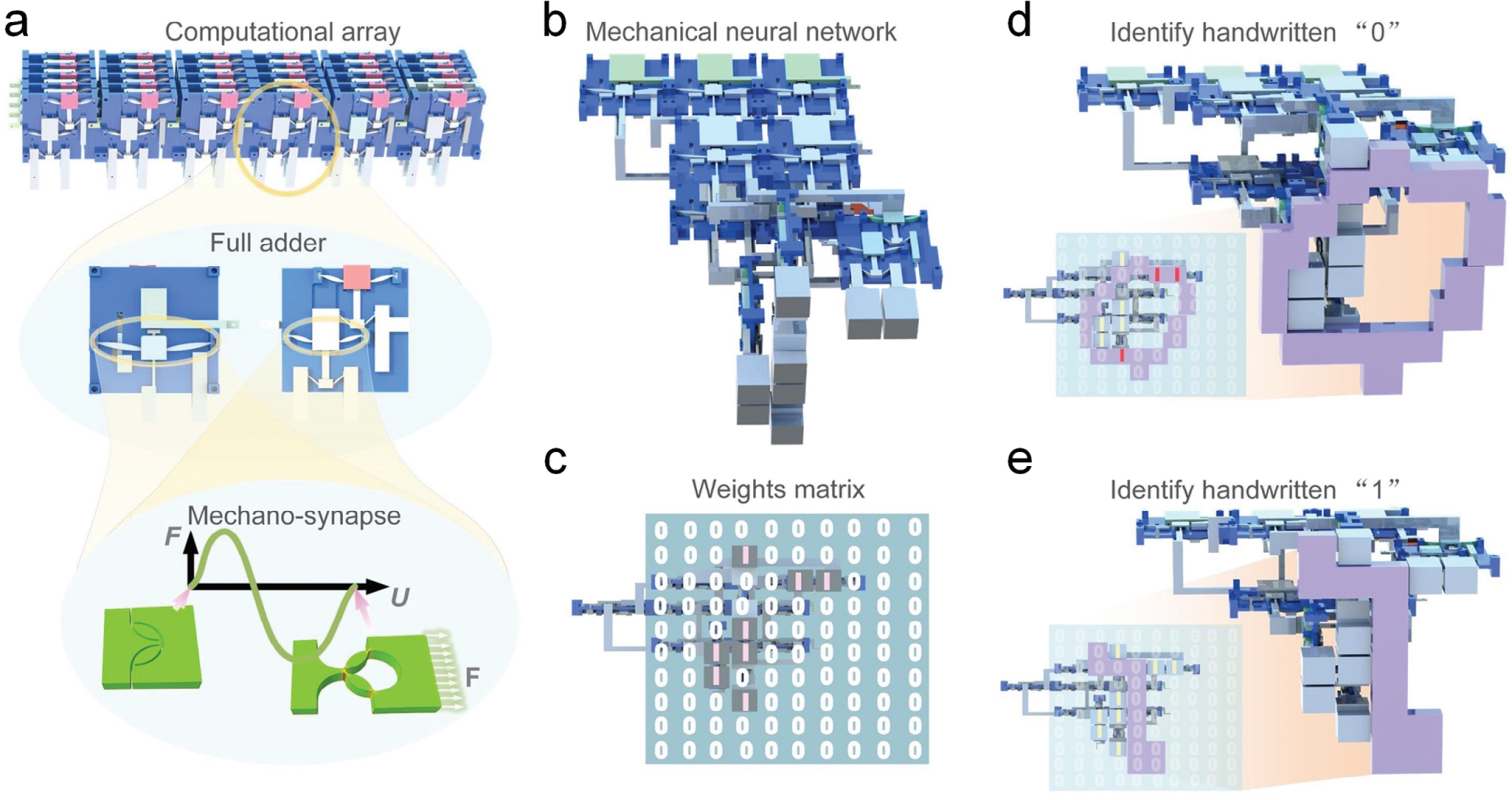
Schematic of a non‐electrical neural network. a) The construction scheme for building a computational array based on a mechanical full adder and mechano‐synapses. b) The structure of the mechanical metamaterial neural network that could identify representations of handwritten “1”s and “0”s. c) The weights matrix of the mechanical neural network. d) The process of the neural network identifying a handwritten “0”. e) The process of the neural network identifying a handwritten “1”.

The concept and functionality of the mechanical neural network are presented in **Figure** **1**. In contrast to the electrical computing system in which semiconductor elements exhibit 0 and 1 states under the application of different voltages, a mechanical bistable petal‐shaped kirigami‐based metamaterial produces 0 and 1 states by applying different mechanical inputs (e.g., force or displacement). Here, the bistable petal‐shaped kirigami pattern (inset of Figure 1a) was chosen as the design basis because it transmits the mechanical signals of both displacement and force, analogous to current and voltage signals in an electrical system. We call this mechanical metamaterial mechano‐synapse similar to a biological synapse that transmits bioelectric signals.

Exploiting the mechano‐synapse concept, we constructed a mechanical computational array with basic calculation functions by connecting mechanical full adders in a series (Figure 1a) to achieve artificial intelligence. The prototype mechanical neural network could distinguish the handwritten “1” and “0” as illustrated in Figure 1b using a specific layout of full adders connected both horizontally and vertically. The mechanical neural network was composed of a series of full adders. The neural network was trained and tested on numerous physical representations of images of handwritten “0”s and “1”s from the Mixed National Institute of Standards and Technology database (MNIST), and the spatial weight matrix of the trained neural network is shown in Figure 1c. The weight matrix of the mechanical neural network was constructed by a 10 × 10 array which has 100 pixels. Because we adopted a binary neural network model, each pixel of the weight matrix could only take the value of 1 or 0. For those pixels that take the value of 0, we regard it as non‐necessary to build the input module, and thus we just keep the pixels that take the value of 1. Different inputs actuated different weight matrix elements of the neural network, and the output was calculated and expressed from the top layer of the system where some snap‐through of the output beams indicated an input of handwritten “0” and others indicated a handwritten “1” (Figure 1d,e). This as‐constructed mechanical neural network demonstrated that very basic intelligence (i.e., recognizing “0” and “1”), could be achieved without any electricity consumption.

### 2.2 Signal Amplification Mechanism of the Metamaterial

To realize the overall aim of this study, namely, to achieve a mechanical neural network as outlined above, obtaining efficient transmission of mechanical signals (force and displacement) was the first crucial challenge. Unlike in electronic systems, the transmission of mechanical signals is difficult to maintain in mechanical systems, primarily because mechanical deformation or force can greatly degrade during propagation between different mechanical parts for various reasons (e.g., friction, material fatigue, tolerance, fabrication error, etc.). To solve this pivotal problem, an amplification mechanism was introduced into the mechano‐synapse.

**Figure 2:**
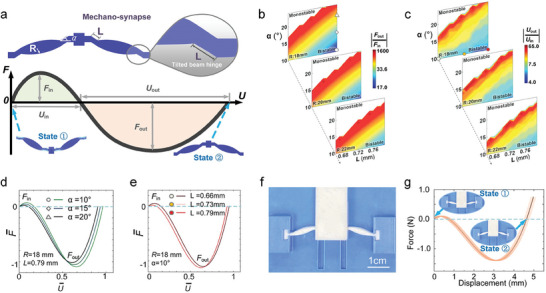
Mechanical signal amplification mechanism. a) The design of a mechano‐synapse and its simulated mechanical performance. b) Simulated phase diagrams of the force amplification factor (output force *F*_out_ over input force *F*_in_) with varying geometric parameters in the mechano‐synapse. c) Simulated phase diagrams for the displacement amplification factor (output displacement *U*_out_ and input displacement *U*_in_) with varying geometric parameters in the mechano‐synapses. d) Simulated mechanical behavior of the mechano‐synapse element for the three different size values marked in the top diagram in b). $\bar{F}$ and $\bar{U}$ are the normalized force and displacement by their maximum value, respectively. e) The simulated mechanical behavior of the mechano‐synapse element for the three different size values marked in the top diagram in c). f) The fabricated mechano‐synapse sample with a force amplification factor of 13.8 and displacement amplification factor of 8.7. Scale bar is 1 cm. g) Measurement results of the fabricated mechano‐synapse sample shown in f). Three samples with the same geometry were fabricated, and the shaded area represents the discrepancies of the three samples, with each tested for five times.

A mechano‐synapse with signal amplifying effect was developed from a typical bistable kirigami‐metamaterial that snaps through^[^ ^25^ ^]^ and consists of two petal‐shaped domains. It is defined by three parameters (*α*, *L*, and *R*), and tilted beam hinges, as shown in **Figure** **2**a. The value range of *α*, *L*, and *R* is determined to enable fabrication accuracy and the size of the synapse (see Methods for detail). Under a certain input of force, this kirigami‐based mechanical metamaterial cycles between two states: state ① as the initial configuration indicating a binary 0 and state ② as the activated configuration representing a binary 1. In terms of force, state ② is a stress‐free configuration, whereas state ① is a force‐free, but not stress‐free configuration. The stress, here, is localized at the tilted beam hinges, connecting the two petals with the main frame. Positive is defined as upward force or displacement, and negative is defined as downward force or displacement. Thus, we are able to perform computing by switching from state ① to ② under certain inputs. We believe progressive computing can be performed by having the outputs from one mechano‐synapse element serve as the input to trigger the next mechano‐synapse. Here, we define and quantify the transmission efficiency as the ratio of output force/displacement to input force/displacement. If the ratio is larger than 1, meaning that the mechanical signal is amplified during the transmission in the mechanical neural network. By contrast, if it is smaller than 1, meaning that the mechanical signal is weakened during transmission.

To ensure efficient transmission of mechanical signals (force or displacement) in progressive computing, the output force and displacement (|*F*_out_| and |*U*_out_|) must be larger than the input (|*F*_in_| and |*U*_in_|) (Figure 2a). To amplify the output force and displacement for a mechano‐synapse, tilted beam hinges with a length of L were introduced to break the symmetry of the bistable structure. Note, before cycling between states, a considerable part of energy can be pre‐stored in the synapse. In Figure 2a, the difference in the output and input areas in the force‐displacement graph is the stored energy in the mechano‐synapse. Then, an amplified mechanical signal can be achieved under the actuation of the pre‐processed synapse.

To determine the relationship between amplification of mechanical signals and geometric sizes (*α*, *L*, and *R*), we used finite element analysis to figure out the relationships between the amplification factors for force (i.e., $\frac{|F_{\mathrm{out}}|}{|F_{in}|}$) and displacement (i.e., $\frac{|U_{out}|}{|U_{in}|}$). The mechano‐synapse geometry parameters are shown in Figure 2b,c. These results indicate that the mechano‐synapse amplification factors vary by several orders of magnitude depending on the geometry of the two petals (*α* and *R*) and the length of the tilted beam hinges (*L*). The resulting force and displacement behaviors for several representative parameter combinations are detailed in Figure 2d,e, which indicate different amplification factors. Note that the largest amplification factor of the mechanical signal might not necessarily be the best choice. While the transmission efficiency of mechanical information must be sufficiently large to enable signal propagation between each of the modules, a sufficient |*F*_in_| is required to prevent unwanted snap‐through interference by environmental vibrations. In other words, a balance between the efficiency of the transmission and robustness against external disturbances should be achieved. We first carried out experiment to test the |*F*_in_| to be at least 0.1 N to maintain the bistable state, which is related to the material adopted to fabricate the sample. Considering the output force equals to the force required to reset the snapped mechano‐synapse, which means larger output force required more external energy to reset the structure. Therefore, we select an appropriate force amplification factor of 15 to balance the signal transmission and resetting energy. To identify the optimum geometric parameters of the mechano‐synapses that satisfies these requirements, a genetic algorithm combined with finite element analysis was applied to determine the best configuration using a fitness function of $f=\sqrt{{\left(|\frac{F_{out}}{F_{in}}|-15\right)}^{2}+1000\cdot {\left(|F_{in}|-0.1\right)}^{2}}$ to search for a $|\frac{F_{out}}{F_{in}}|$close to 15, and |*F*_in_| close to 0.1 N, which is sufficient to resist the surrounding disturbance (see Figure S1, Supporting Information).

We achieved an optimized mechano‐synapse with a force amplification factor of ≈12.8 and a |*F*_in_| of ≈0.1 N. A mechano‐synapse with these parameters was fabricated using 3D printing (Figure 2f), and its mechanical behavior in terms of force versus displacement was then tested (Figure 2g). Compared with the predicted performance (Figure S1, Supporting Information), the 3D printed prototype showed similar behavior, with an amplification factor of 13.8, which is sufficient to transmit mechanical signals efficiently in different kirigami modules. Additionally, the required |*F*_in_| of ≈0.1 N maintained a relatively robust snap‐through state.

### 2.3 Design of the Full Adder

**Figure 3:**
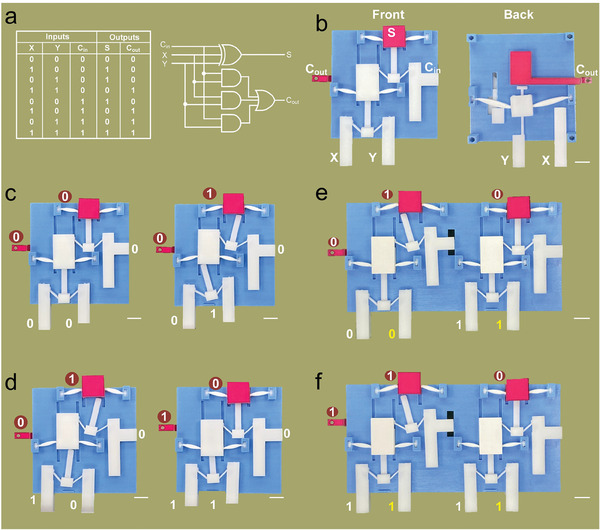
The top‐down design approach of a mechanical full adder. a) Truth value table of a full adder and its electrical circuit expression. b) Design and the photo of the front and back sides of the mechanical full adder. c) Calculation process of a binary addition of 0 + 0 and 0 + 1 by a one‐bit mechanical computing unit. d) Calculation process of a binary addition of 1 + 0 and 1 + 1 by a one‐bit mechanical computing unit. e) Calculation process of a binary addition of 01 + 01 by a two‐bit mechanical computing array. f) Calculation process of a binary addition of 11 + 11 by a two‐bit mechanical computing array. The scale bar in b–f) is 1 cm.

We first implemented our mechano‐synapse in basic logic gates (i.e., AND and OR gates) and then more complicated gates (i.e., XOR gate, SR latch, and half adder), as shown in Figures S2,S3 (Supporting Information), respectively, and Video S1 (Supporting Information). This was achieved before starting the next and more complex task of constructing a mechanical full adder. The full adder design was challenging because it had eight different combinations of inputs and outputs (**Figure** **3**a), and its corresponding electronic bottom‐up design had three AND gates, one 3‐input‐OR gate and one 3‐input‐XOR gate with intricate connections. Thus, it was relatively difficult to construct the full adder by directly following its electrical circuit. These connections therefore obstructed our ability to construct the mechanical full adder using the traditional bottom‐up design used in electric systems. Instead, a top‐down method was used to arrange the mechano‐synapses for satisfying the force/displacement amplification and minimum |*F*_in_| requirements for meeting the desired combinations in the truth value table.

The resulting design was a mechanical full adder that added three inputs (i.e., *X* and *Y* as the operands and *C*_in_ as a bit carried in from the previous calculation) and generated two outputs (i.e., summary bit *S* and the output carry *C*_out_), as shown in Figure 3b. The front side and the back side of the full adder comprised different layouts of mechano‐synapses, which were designed independently in a top‐down manner. The as‐designed full adder only required three logic gates (2 XORs for the front side and 1 3‐input gate for the back side), ensuring a relatively simple configuration of the full adder for fabrication purposes. The front side XOR gates were connected in a series to calculate the output summary bit S, thus functioning similarly to a 3‐input‐XOR gate (Figure S4a, Supporting Information). The back side worked to obtain the output carry bit *C*_out_. When the number of actuated parameters (*X*, *Y*, *C*_in_) is larger than two, *C*_out_ is 1; otherwise, *C*_out_ is 0 (Figure S4b, Supporting Information).

Figure 3c,d presents four different inputs and outputs for a mechanical full adder, which corresponds well with the truth value table. More complex calculations with higher bits are readily achievable by connecting multiple full adders in a series. For example, two connected mechanical full adders successfully calculated 01 + 01 = 010 (Figure 3e) and 11 + 11 = 110 (Figure 3f). Additional details about the full adder can be found in Figure S4 and Video S2 (Supporting Information), including the assembly process, as well as more calculation configurations with different inputs/outputs. The full adder can be reset by resetting a plate, as shown in Figure S4i,j, and Video S3 (Supporting Information). More complex assemblies of full adders were then used to construct the mechanical neural network.

### 2.4 Construction of the Non‐Electrical Neural Network

**Figure 4:**
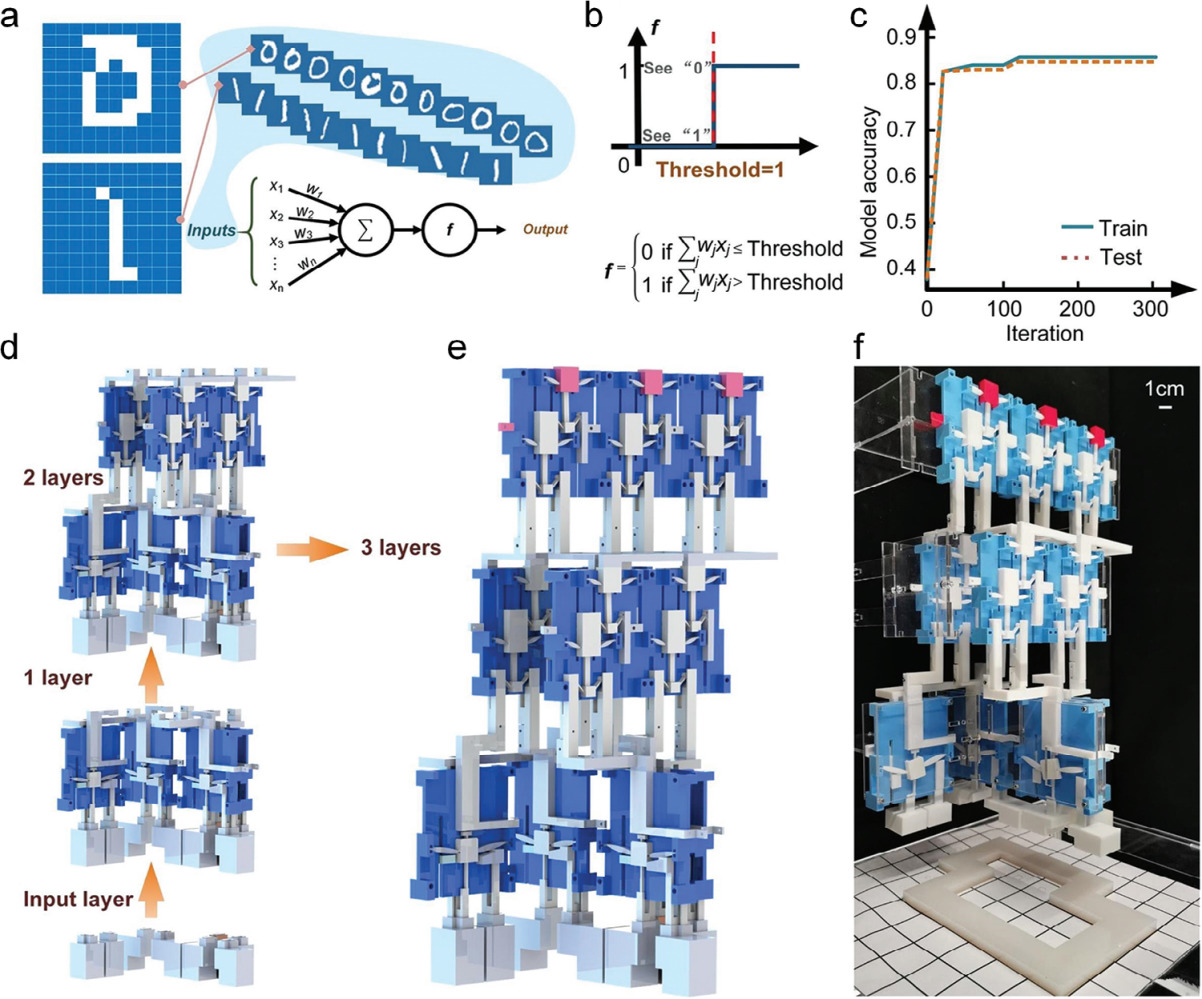
Non‐electrical neural network for identification of handwritten “0”s and “1”s. a) Schematic of the neural network and the data used to train the model. b) The nonlinear function adopted by the neural network model. c) The training and testing process for the neural network model. d) The construction strategy that builds the mechanical neural network layer by layer to add up all the input values from the weight matrix. e) The established 3‐layered mechanical neural network model. f) The fabricated mechanical neural network.

Based on the design of the kirigami‐based modules, namely, a full adder that can efficiently transmit mechanical signals to conduct computation, we built a mechanical neural network to demonstrate a nonelectrical mechanical computing system for image recognition functionality (**Figure** **4**). A binary neural network that quantitatively recognized handwritten images of “0”s and “1”s was adopted to generate a simple model with a weight matrix composed of only 0 or 1. A dataset of handwritten images of “1” and “0”, obtained from MNIST and as shown in Figure 4a, was used for training and testing of the neural network. The weight matrix was obtained by computationally training a binary neural network by MNIST, and then we used the trained weights to physically construct the logic gates to realize the computational functions of the neural network. Each image for training was pixelized into a 10 × 10 array with the color of pixels either black or white, which for the full adder would be an input of 1 or 0, respectively.

The nonlinear function of the binary neural network is shown in Figure 4b, where *x*_j_ = 1 or 0 represents the input value (push‐in for 1 or not for 0) and *w*_j_ = 0 or 1 is the corresponding weight factor for the binary neural network model. The neural network was trained to provide an output for identifying a handwritten “0” if the calculated summation $\sum_{j}w_{j}x_{j}$ exceeded a threshold of 1. In other words, if the output of a full adder was larger than 1, the neural network would tell us it “recognizes” an image of the handwritten “0”. If $\sum_{j}w_{j}x_{j}\leq 1$, it would tell us it “recognizes” an image of a handwritten “1”. The constructed weight matrix was composed of eight 1s and ninety‐two 0s (Figure 1c), meaning the maximum value of the calculated summation was eight. Therefore, to calculate any possible input combinations, a three‐layered mechanical neural network with each full adder performing pairwise addition was required to achieve the mechanical neural network.

To ensure the transmission efficiency of mechanical signals across different modules, a carefully designed topology of the multiple kirigami‐based mechanical metamaterial system was critical. Thus, we adopted the principle of proximity (using as minimum connections as possible to connect the full adders) for arranging the full adders in space. This involved constructing the mechanical neural network layer by layer in the most compact manner possible (Figure 4d,f; Figures S5–S7, Supporting Information provide details). The handwritten image models (“0” or “1”) to be recognized were pressed against the bottom of the mechanical neural network, which actuated the corresponding cubes in the input layer and then progressively transmitted mechanical signals through the next two layers via the embedded logic in the full adders. The result was represented by red output blocks on the top layer of the system. The larger the calculated value, the closer the handwriting model was to a standard “0”. Therefore, any actuation of the three red cubes indicated a handwritten image of “0”, while no actuation indicated a handwritten image of “1”. The constructed 3‐layer mechanical neural network model trained on this method possessed a prediction accuracy of 0.83 on the testing set (Figure 4c).

### 2.5 Demonstration of the Non‐Electrical Neural Network Performance

The performance of the mechanical neural network was tested using pixelized handwritten “1”s and “0”s, also taken from the MNIST database. To physically verify the identification accuracy of the mechanical neural network, we fabricated 10 different 3D printed models of handwritten images of “1” and “0” in each. All the printed models were bonded onto a transparent plate so that we could easily record the image recognition process in photos and videos. The input handwritten plates were then pushed against the bottom layer of the mechanical neural network to execute the image recognition algorithm (see Video S4 (Supporting Information) for the recognition of “0” and Video S5 (Supporting Information) for the recognition of 3 representative “1”). Figures S8–S10 (Supporting Information) show the detailed functioning process of the mechanical network to identify a handwritten “0” and “1”, featuring some local views of the mechanical neural network system. After testing all these 3D printed models, the results showed that 100% of “1” models provided a correct binary calculation summary of 0000 because none of the eight input pixels based on the weight matrix were actuated. Then 90% of “0” models correctly led to a binary calculation summary larger than 1 (i.e., 0011) because more than 1 input pixels were actuated.

To verify the strong adaptability of a full adder to the environment, we also conducted experiments in low temperatures after letting the system in ‐20 °C environment for >24 h (Video S6, Supporting Information). This video shows that the full adder maintained its accurate calculation function. By contrast, the electrical calculator cannot function in this low temperature. It should be noted that extreme environmental conditions include various aspects, such as high radiation, large temperature changes (high and low temperatures), low pressure, zero gravity, and so on. Here, we only considered the low temperature as a demonstration of extreme temperature conditions due to the limitation of experimental conditions. From a large number of physical tests, we were able to conclude that the mechanical neural network demonstrated strong robustness and reliability for recognizing 3D printed representations of handwritten “0” and “1” images.

## 3 Conclusion

This paper presented a non‐electrical mechanical computing system constructed using purely mechanical elements with concise topological architectures and efficient transmission of mechanical signals. The system was designed via a top‐down methodology which allowed for simplified fabrication. Though very simple intelligence is demonstrated, this work represents a viable method of combining multiple functional kirigami‐based metamaterials that can be further developed toward building a fully functional mechanical computing system. It is also expected that more complicated mechanical neural network (e.g., more hidden layers, different activation functions) with sophisticated functionalities can be constructed by combining with advanced fabrication methods (e.g., microfabrication for microscale components to enable high‐density assembly) and smart materials (e.g., light‐sensitive materials for light trigged computing and recognition). In essence, this study opens the door to discovering more complex versions of mechanical computing systems. We demonstrated at a very basic level that computing, learning, and even forgetting can emerge from this unexplored area of non‐electrical computing systems. At present, the weight value in our work can only be taken from 0 and 1, which limits the functionality and accuracy of the mechanical neural network. The ideal effect is to design a neural network model that could classify all handwritten numbers from MNIST. In this situation, the physical construction of the mechanical neural network would be far more complex than its current version. In the future work, more sophisticated design methodology will be studied to achieve more complicated functions. A recently reported work demonstrates the advantage of achieve programmable weight value implemented by a machinery system,^[^ ^26^ ^]^ which indicates the trend to enrich the weights of neural networks to achieve more complex and accurate functions despite of its more intricate assembly. Future efforts will be made to balance the accuracy of the mechanical computing and the complexity of the mechanical system.

## 4 Experimental Section

### Optimization of the Kirigami Metamaterial

To identify the geometric parameters of the mechano‐synapses that satisfied the requirements of both mechanical signal amplification and robustness against accidental induction of the snapped‐through state, a genetic algorithm combined with finite element analysis was applied using the fitness function ($f=\sqrt{{\left(|\frac{F_{out}}{F_{in}}|-15\right)}^{2}+1000\cdot {\left(|F_{in}|-0.1\right)}^{2}}$) (see Figure S1, Supporting Information). The structural evolution process was performed to find out the optimal meta‐atom. Here, the genetic algorithm (GA) model was used as the machine learning method. GA could be readily combined with the COMSOL Multiphysics finite element simulations via LiveLink for MATLAB. Details could be found in the literature.^[^ ^23^ ^]^ By applying GA, a population size of 30 mechano‐synapses was initially generated. Then, the genetic algorithm was employed to find out an optimal solution by genetically breeding a population of individuals over a series of generations until the change of fitness function between two adjacent generations was smaller than the tolerance.^[^ ^27^ ^]^ Based on fitness values, the sort and perform roulette wheel selection operations on the population, choosing individuals with higher fitness to proceed to the next generation. Then, crossover and mutation operations was applied to generate new individuals. The crossover operation was generated new offspring individuals by combining parts of the features of two‐parent individuals. The mutation operation introduces new variations by randomly changing some features of an individual. To avoid premature convergence and being trapped in local optima, it was set the crossover and mutation probabilities to 85% and 20%, respectively. The maximum iteration generation was 200. The mechanical behavior of the 3D printed part was then tested and had a force amplification factor of ≈13.8 and required |*F*_in_| of ≈0.1 N depending on the materials it was used (TPU) and the fabrication accuracy.

### Mechanical Measurements of Mechano‐Synapses

The mechanical performance of the mechano‐synapses shown in Figure 2g was tested by a Universal material testing machine (Instron, USA). The mechano‐synapses were snapped to state ①, then, a predetermined displacement was applied on the middle beam of the structure to snap back the structure to state ②. The loading velocity was 1 mm min^−1^. Three samples of the optimal mechano‐synapse were fabricated. Each sample was measured for three times.

### Finite Element Method (FEM)

The finite element analysis results shown in Figure 2b,e are calculated by ABAQUS (Dassault Systèmes, France) and the petal‐like structure had a Young's Modulus of 500 MPa, and Poisson's ratio of 0.3, which corresponded to the mechanical parameters of TPU used in 3D printing process. The model was meshed using hybrid eight‐node linear brick elements and mesh sensitivity analysis was conducted to ensure numerical convergence. To calculate the amplification factors of the mechano‐synapse with different combinations of geometric size, a Python script was used to iterate through the parameters and the data of force‐displacement curves were saved in the form of .txt. After all calculations were finished, the results were then processed to obtain Figure 2b,c.

The finite element analysis software COMSOL Multiphysics 5.6 was employed for the optimization of mechano‐synapse shown in Figure 2b‐d. By incorporating the solid mechanics module and the steady‐state solver, it was successfully established a simulation model and calculated the force–displacement curve. During the modeling process, it is discretized the structural domain using triangular elements. To evaluate the performance characteristics of the bistable structure, it was extracted the snap‐through force as a key indicator from the force–displacement curve.

### Construction of Basic Logic Gates

Different logic gates and calculation modules were constructed based on bistable kirigami‐based metamaterials. Mechanical AND and OR logic gates were executed through the two‐petal kirigami metamaterials by limiting the push‐in deformation. This enabled the use of a whole family of common logic gates with similar structures. Figure S2 (Supporting Information) presented three basic logic gates, namely, the AND gate (Figure S2a–d, Supporting Information), OR gate (Figure S2e–h, Supporting Information) and NOT gate (Figure S2i,j, Supporting Information). Finite element analysis simulations were carried out using ABAQUS to obtain the results of the stresses on the mechano‐synapse. The finite element results illustrated that the stress was localized at the hinges of the two petals, while the rest of the structure was generally stress‐free.

More complicated logical gates and calculation modules were shown in Figure S3 (Supporting Information). The XOR gate (Figure S3a, Supporting Information) was designed by a top‐down method (i.e., focusing on achieving the truth value table), consisting of two complementary blocks that plug into each other when aligned. For a “0” output, two inputs take the same value (both “0” or both “1”), and thus, these two blocks align and prevent actuation of the output. Otherwise, the two blocks contact each other, and the output was actuated for a “1” state. A model for a mechanical version of a frequently used writing and storing module, SR latch, was also designed in a top‐down manner (Figure S3b, Supporting Information). In this model, the state of *Q* can be controlled (or “written”) by *S* only when *R* was not actuated. Otherwise, when *R* was actuated, the value of *Q* was reset regardless of the state of *S*. The design based on the top‐down method is much simpler than that based on the bottom‐up method. This function is achieved by the designed mechanical structure when the boundary condition is changed by the *R* module. When the *R* module was actuated, the right‐side boundary of *Q* module was weakened, which could not support the bistable performance of *Q*. Therefore, the *Q* module was back to its initial state when *Q* = 1. A half adder was also conceived (Figure S3c, Supporting Information), in which the summary output *S* was obtained by an XOR gate, and the carry‐out *C* was derived from an AND gate.

### Layout of the Non‐Electrical Neural Network

To ensure the efficient transmission of mechanical signals (force and displacement) between the full adders that make up the mechanical neural network system, it was necessary to determine the most concise layout of the full adders in each layer. A principle of proximity was adopted to determine the best arrangement. Here, the distance between two adjacent pixels was set to 1, and the conciseness factor *Q*, defined as the sum of the distance between each connected full adder, was used to quantify the conciseness of the topological arrangement. Figure S5 (Supporting Information) lists six possible connection schemes for the first layer of the mechanical neural network being applied to a weight matrix with a maximum of eight 1s with the required number of individual full adders to perform four pairwise additions. Here, topology ① was the most concise layout with the minimum conciseness factor *Q* = 11. The same assessment method was adopted for the other layers of the mechanical neural network. The details and the most concise layout of the second and third layers of the mechanical neural network were given in Figures S6,S7 (Supporting Information), respectively, where A, B, C, D represent full adders installed on the input plate, and A^T^, B^T^, C^T^, D^T^ represent the corresponding full adders rotated by 180°. Based on the corresponding position according to the most concise topological arrangement, the first layer of the mechanical neural network composed of four single full adders could be installed separately. Then, the second layer of the mechanical neural network composed of two sets of two full‐adder conjunctions and the third layer of the mechanical neural network with a set of three full‐adder conjunctions could be constructed and installed. To ensure the construction accuracy of the mechanical neural network, connection beams were appropriately designed and fabricated by 3D printing to firmly join the inputs and outputs between adjacent layers. ABS (Acrylonitrile Butadiene Styrene) materials was used for the connection beams to minimize the deformation and ensure the transmission efficiency of mechanical signals between each layer.

### Prototyping—Logic Gates

The frameworks of the logic gates were fabricated by material jetting 3D printing (J826 Prime, Stratasys, USA) with VeroWhite resin. The white flexible petal‐shaped structure was fabricated by fused deposition modeling (FDM) 3D printing (E2, Raise3D, China) with TPU (PolyFlex TPU 95) materials. After fabrication, the petal‐shaped structure was installed in the framework and could be snapped through under an external force.

### **Prototyping—**Full Adder and the Non‐Electrical Neural Network

The blue frameworks of the full adder and mechanical neural network were fabricated by material jetting 3D printing (J826 Prime, Stratasys, USA) using VeroWhite resin. The white petal‐shaped structure was fabricated by FDM 3D printing (E2, Raise3D, Shanghai) with TPU (PolyFlex TPU 95) materials. It should be noted that the petal‐shaped structure could also be fabricated by laser cutting for more efficient and low‐cost fabrication. The input modules could be pushed onto the input area of the full adders to actuate them. The connection beam between each layer of the mechanical neural network was fabricated by FDM 3D printing (E2, Raise3D, Shanghai). To ensure high signal transmission efficiency of the force and displacement, it was used the rigid material eSilk‐PLA (eSUN, USA). Small screws were used to fasten different sections of the connection beams. All the full adders in different layers of the mechanical neural network were installed on and fixed by a transparent acrylic plate to ensure robustness.

### **Prototyping—**Handwritten “1” and “0” Models

The handwritten images of “0” and “1” from the dataset of MNIST to test the constructed mechanical neural network. Representations of 10 handwritten “1” and “0” each were fabricated by laser cutting (CMA0604‐G‐R, Han's Yueming Laser, China) with acrylic plate. Each handwritten image was then bonded together with a pixelized transparent plate fabricated with size of 256 mm × 256 mm × 10 mm.

## Conflict of Interest

The authors declare no conflict of interest.

## Supporting information

Supporting Information

Supplemental Video S1

Supplemental Video S2

Supplemental Video S3

Supplemental Video S4

Supplemental Video S5

Supplemental Video S6

## Data Availability

The data that support the findings of this study are available from the corresponding author upon reasonable request.
