# Supplementary material for: Mechanical Metamaterials for Handwritten Digits Recognition
Source: Adv Sci (Weinh). 2023 Dec 25;11(10):2308137. doi: 10.1002/advs.202308137 (PMC10933649; doi:10.1002/advs.202308137)
Supplement: Supplementary file 1 — Supporting Information [file ADVS-11-2308137-s005.pdf]

ADVANCED SCIENCE Open Access

Supporting Information

for *Adv. Sci.*, DOI 10.1002/advs.202308137

Mechanical Metamaterials for Handwritten Digits Recognition

*Lingling Wu, Yuyang Lu, Penghui Li, Yong Wang, Jiacheng Xue, Xiaoyong Tian, Shenhao Ge, Xiaowen Li, Zirui Zhai, Junqiang Lu, Xiaoli Lu, Dichen Li and Hanqing Jiang**

Supporting Information

Mechanical Metamaterials for Handwritten Digits Recognition

*Lingling Wu†, Yuyang Lu†, Penghui Li, Yong Wang, Jiacheng Xue, Xiaoyong Tian, Shenhao Ge, Xiaowen Li, Zirui Zhai, Junqiang Lu, Xiaoli Lu, Dichen Li, and Hanqing Jiang**

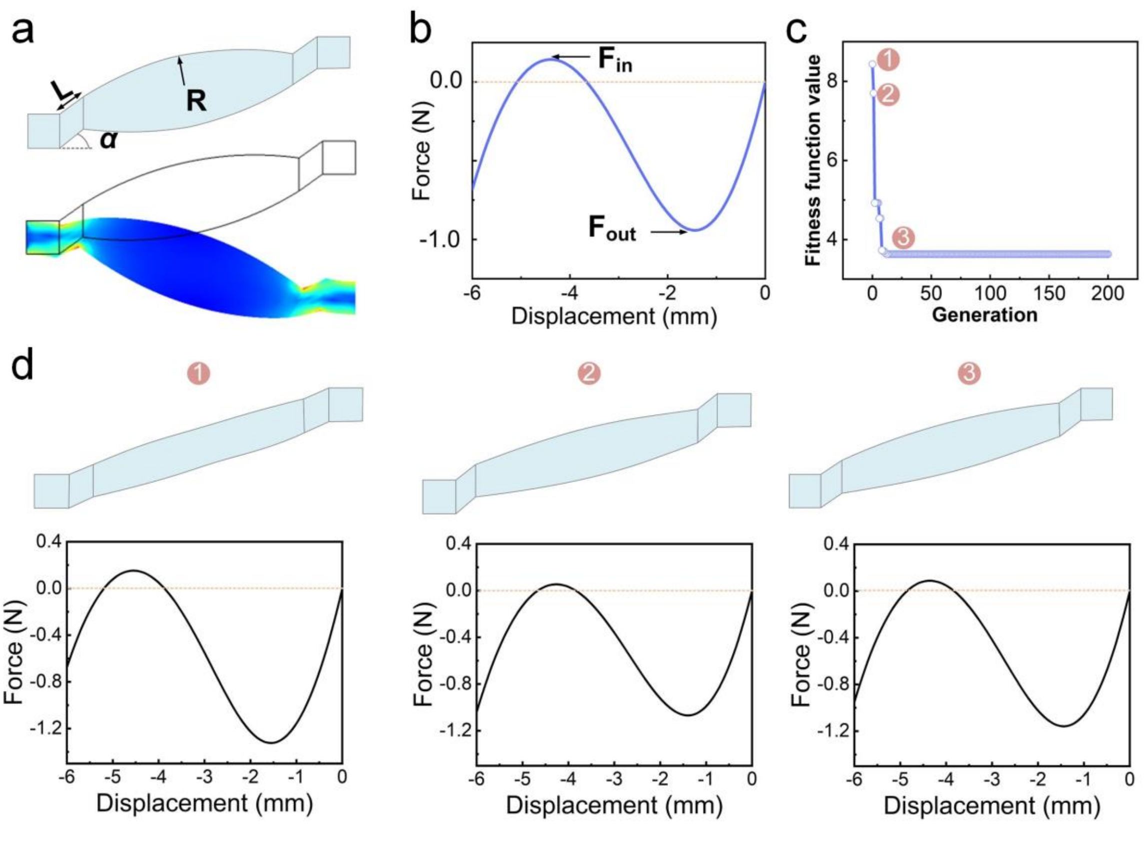

Figure S1. Optimization process of the mechano-synapses. a) The basic structure of the mechano-synapses and their key geometric size parameters. b) The mechanical behavior of a typical synapse. c) The evolution of the search for the optimum mechano-synapses with the

optimum $|F_{out}/F_{in}|$ and $|F_{in}|$ with by a genetic algorithm. d) The mechanical behavior of three mechano-synapses with different configurations marked in c).

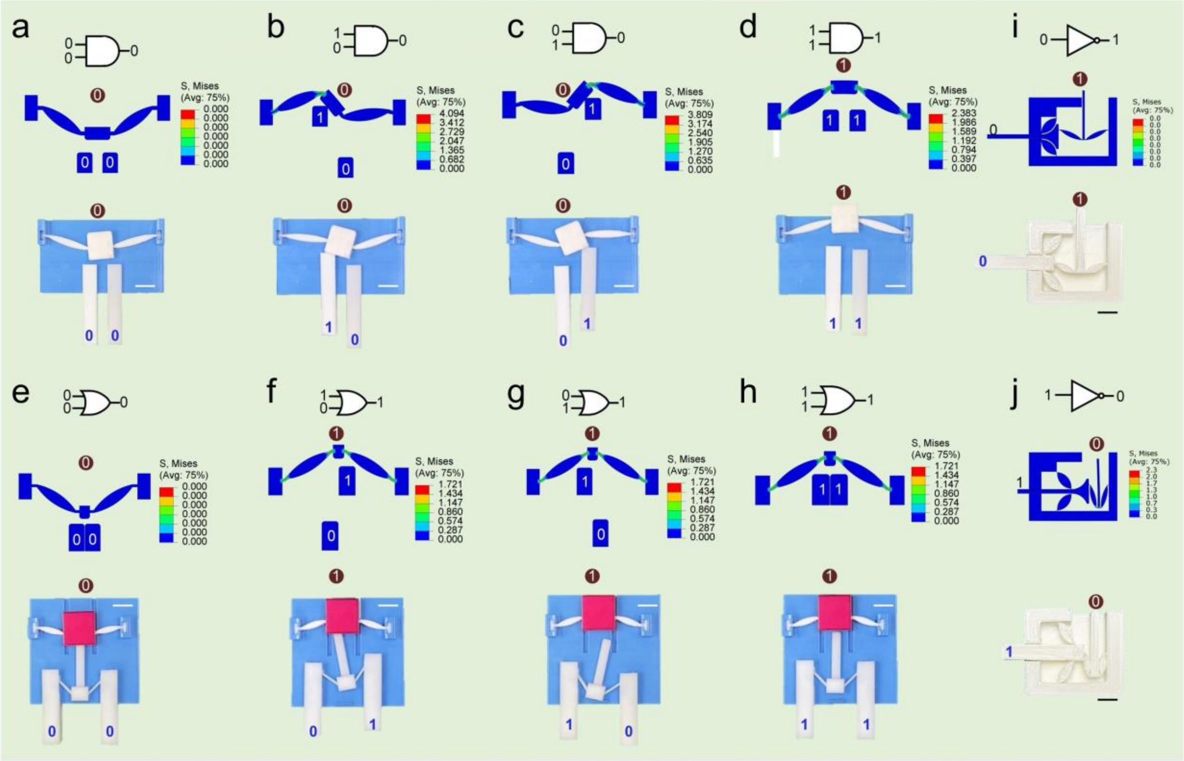

Figure S2. Design of basic mechanical logic gates. (a-d) Simulated mechanical behavior of an AND gate for different inputs. (e-h) Simulated mechanical behavior of an OR gate for different inputs. (i-j) Simulated mechanical behavior of a NOT gate for different inputs. The corresponding fabricated samples are presented below each simulated configuration. The scale bar in (a-j) is 1 cm.

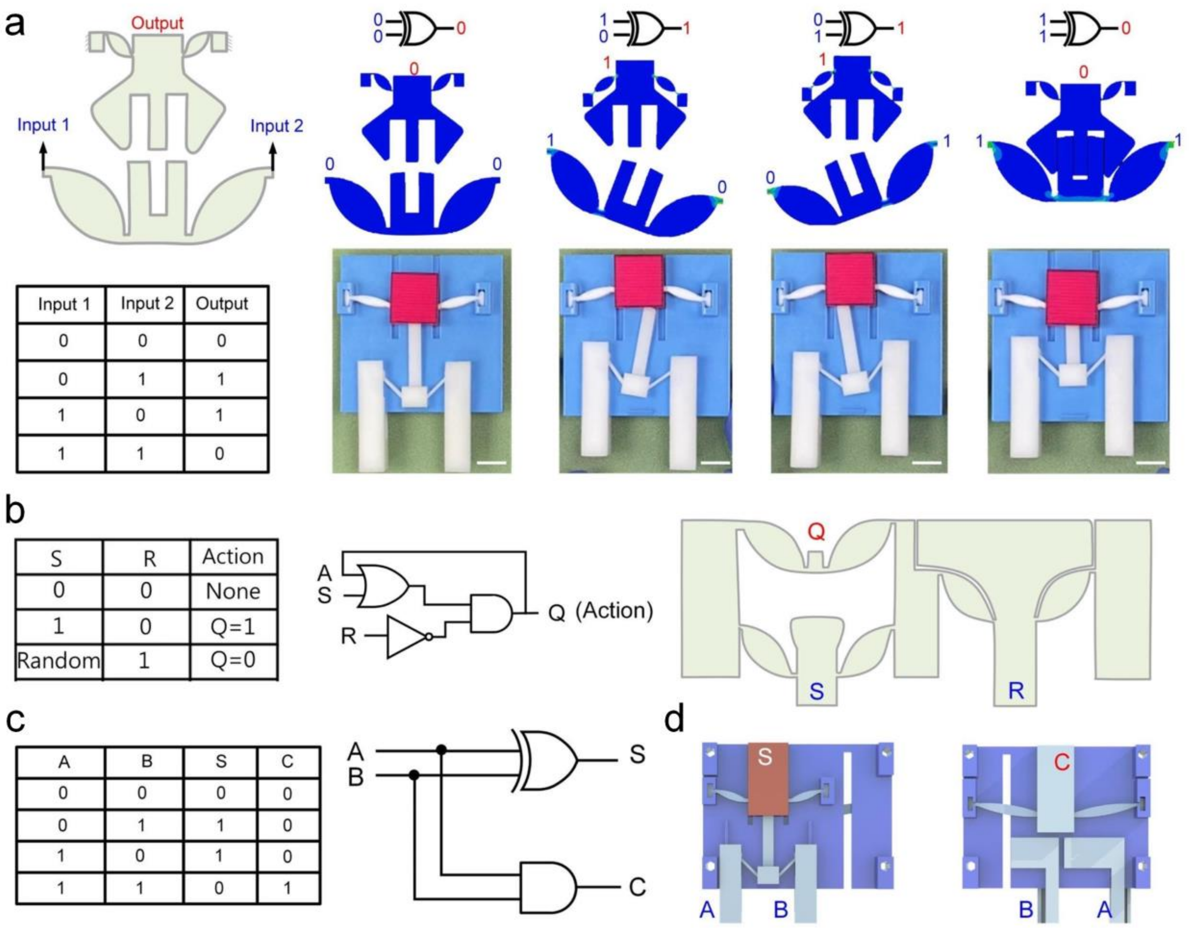

Figure S3. Top-down design process of some common mechanical logic modules. a) The designed and fabricated XOR gate model and its functions with different input values. b) The designed SR latch model. (c-d) The designed and fabricated half adder model and its truth table with different input values. The left and right panels of d) are the front and back sides of a half adder, respectively. The scale bar in a) is 1 cm.

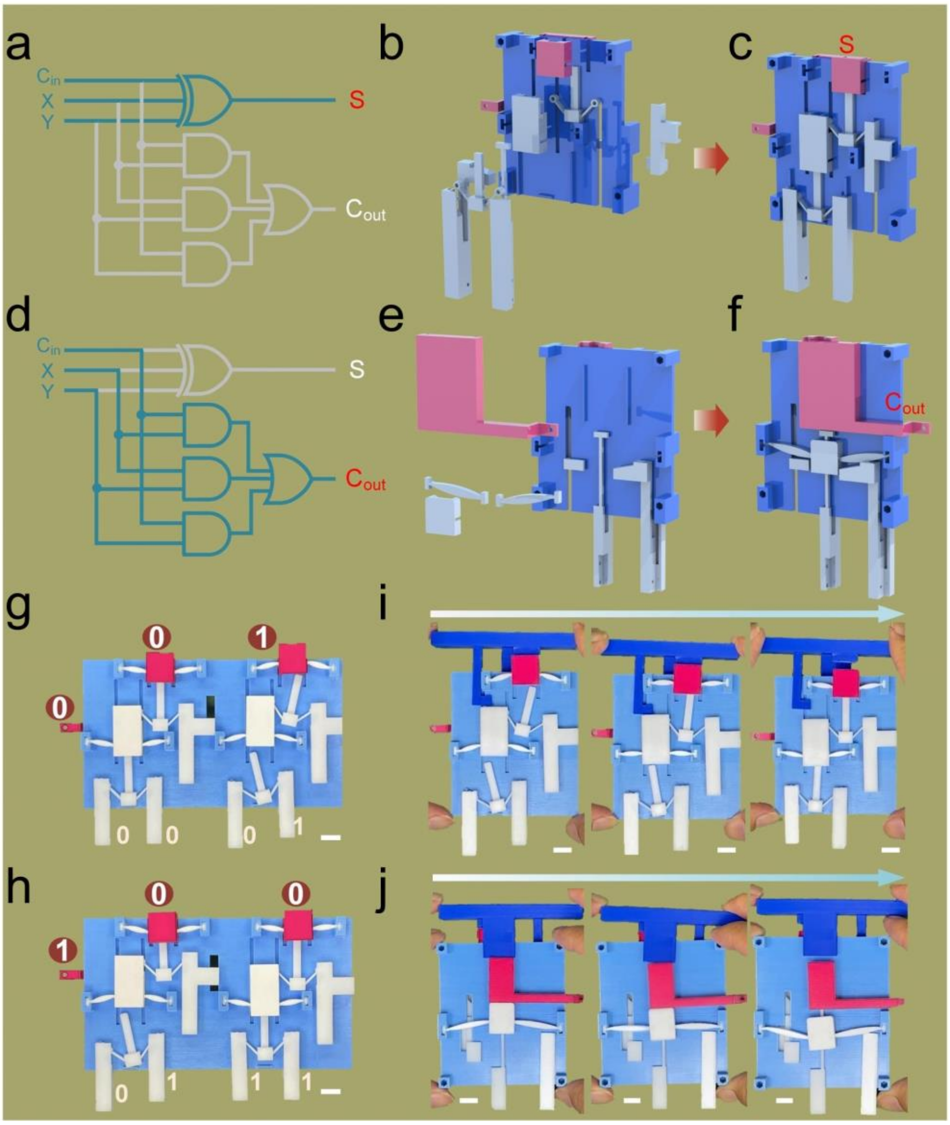

Figure S4. Details of the full adder model. a) The logic circuit of the front side of a full adder. b) An exploded view of the front side of a mechanical full adder model. c) The front side of a designed mechanical full adder. d) The logic circuit of the back side of a full adder. e) An exploded view of the back side of a mechanical full adder model. f) The back side of a designed mechanical full adder. g) Two fabricated mechanical full adders as a two-bit mechanical computing array calculating 00+01. h) Two fabricated mechanical full adders as a two-bit mechanical computing array calculating 01+11. (i-j) The reset process of the full adder from the front view i) and back view j), in which the input and output beams are pushed back by the resetting plate simultaneously. Automatic reset could also possibly be achieved by introducing

elastic components between the input beam and the framework so that the elastic parts could store part of the mechanical energy and release it after the input actuation is unloaded. The scale bar in (g-j) is 1 cm.

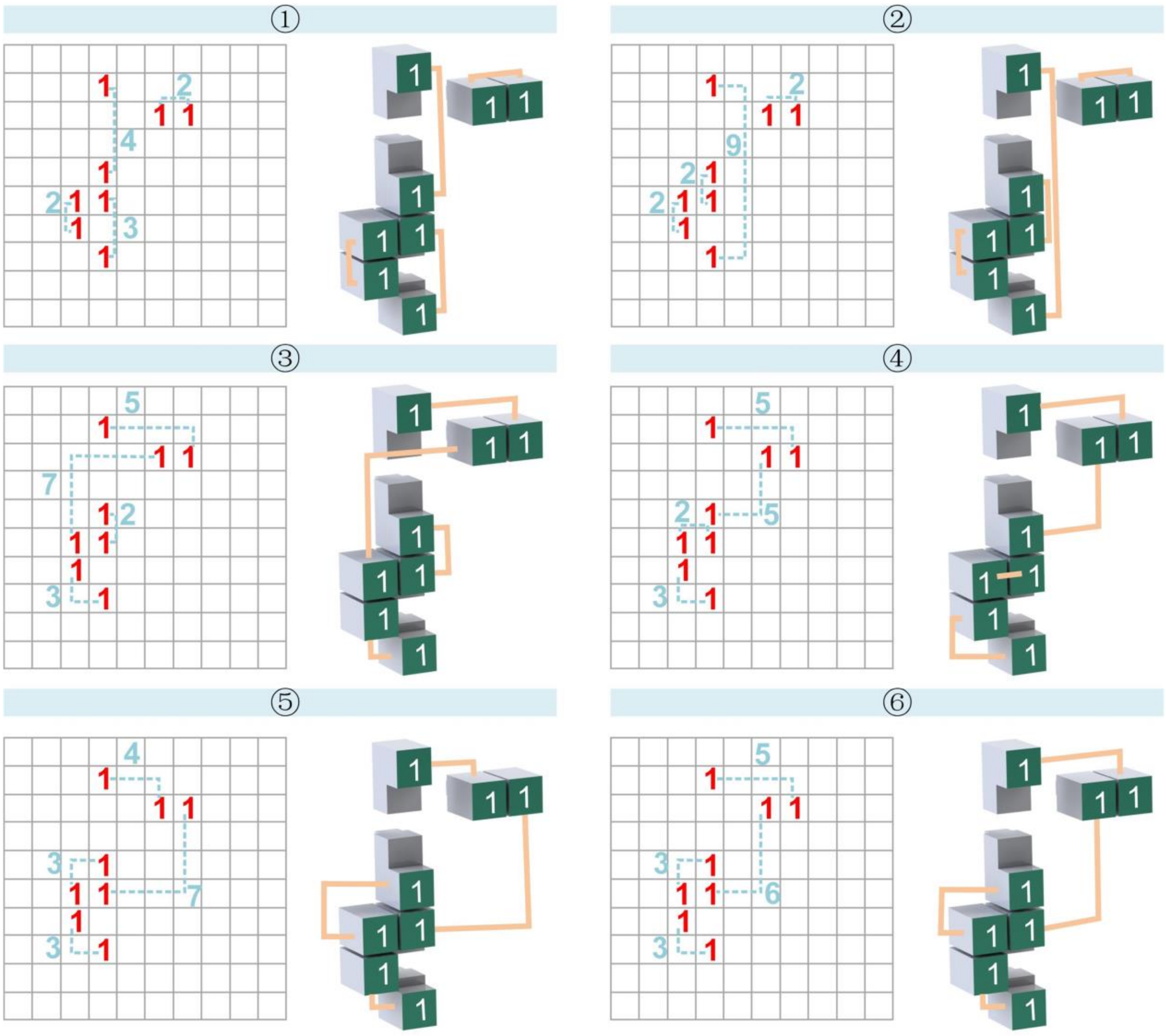

Figure S5. Possible layouts of the mechanical neural network first layer. Here, the distance between two adjacent pixels was set to 1, and the conciseness factor Q, defined as the sum of the distance between each connected full adder, was used to quantify the conciseness of the

topological arrangement. The dash lines in the matrix represent the beam that we need to connect the corresponding inputs by a full adder.

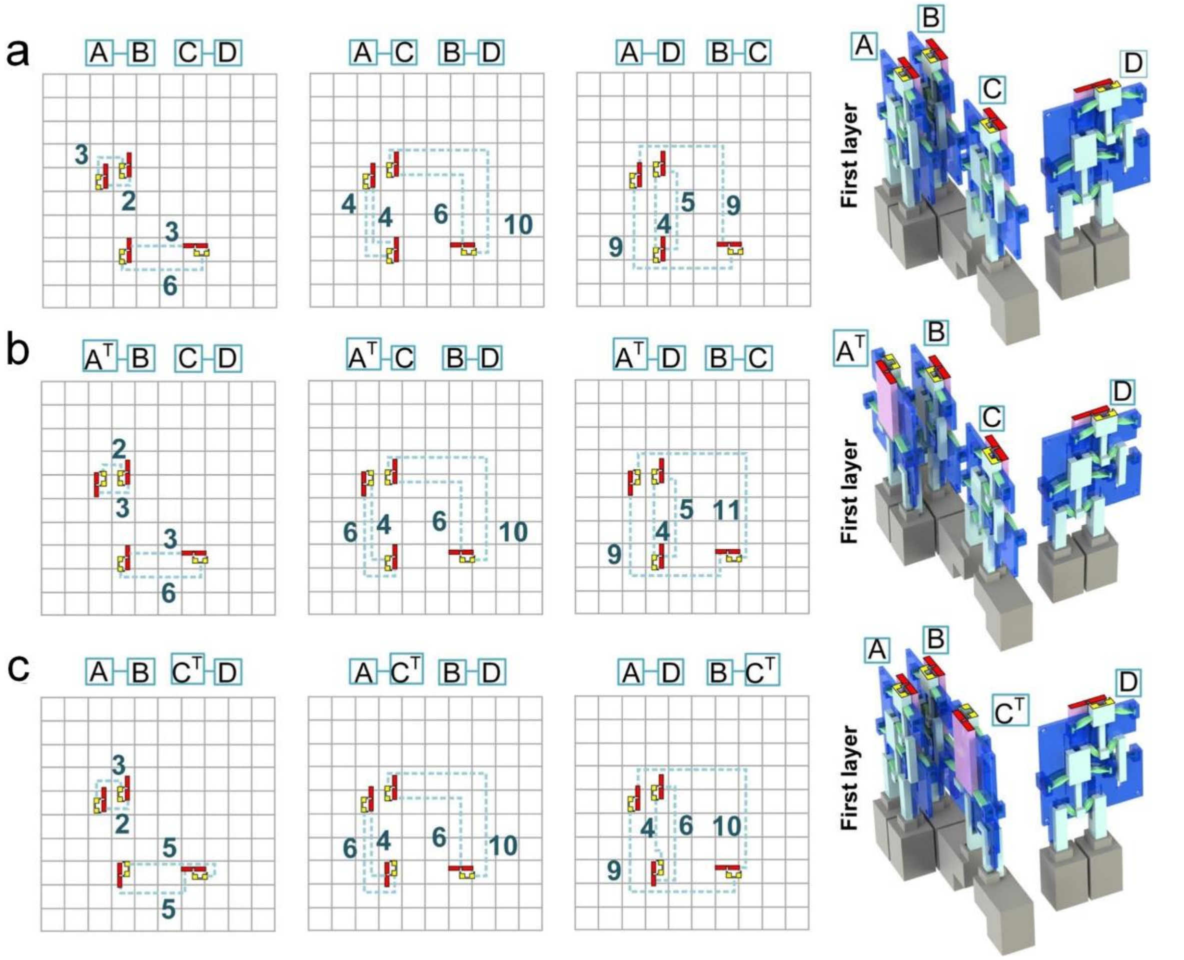

Figure S6. Possible connection plans of the mechanical neural network second layer. The sum bit is in yellow and the carry-out bit in the weight matrix is in red. The dash lines in the matrix represent the beam that we need to connect the outputs of the full adders in the first layer, so that they could be applied as the inputs for the full adders of the second layer.

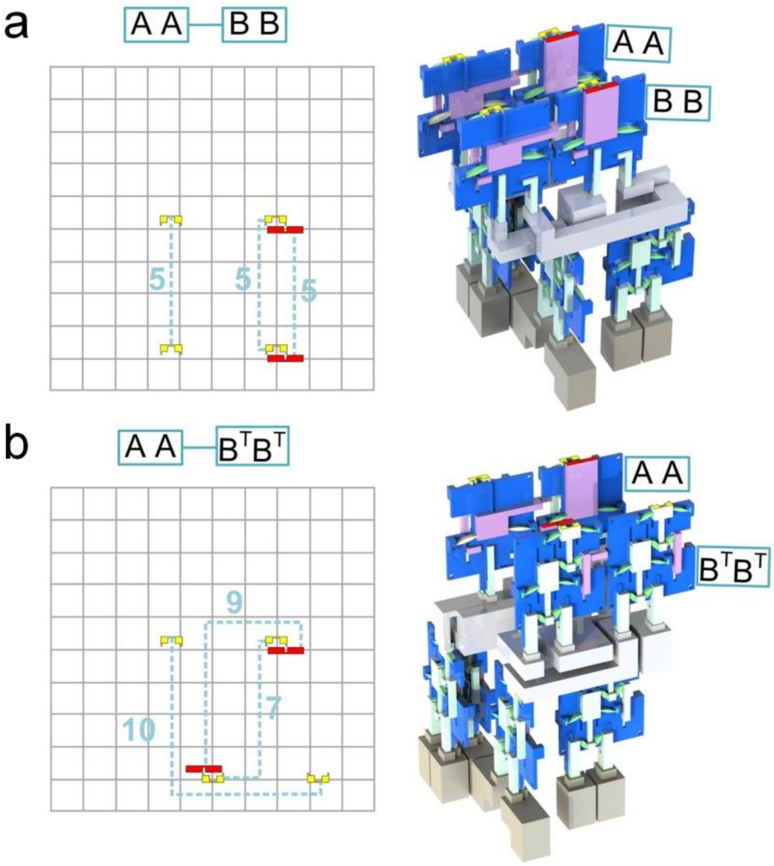

Figure S7. Possible connection plans of the mechanical neural network third layer. The sum bit is in yellow and the carry-out bit in the weight matrix is in red. The dash lines in the matrix represent the beam that we need to connect the outputs of the full adders in the second layer, so that they could be applied as the inputs for the full adders of the third layer.

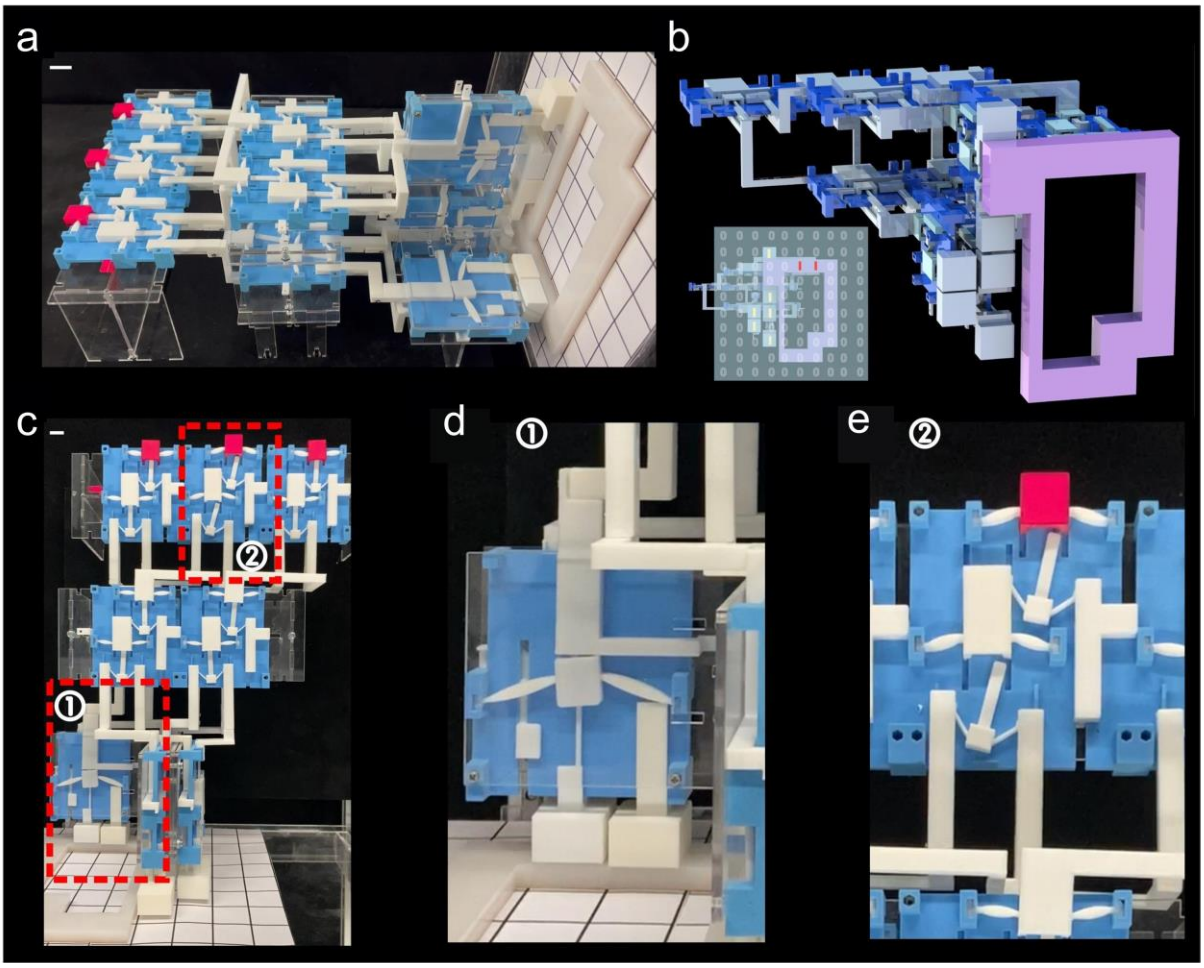

Figure S8. Functionality of the mechanical metamaterial to identify a handwritten “0”. a) Photograph of the fabricated metamaterial system and its functional process to identify a 3D printed handwritten “0”. b) The weight matrix pixels that were actuated by the handwritten “0”. c) A front view of the metamaterial system. d) Local view of the ① component marked in c). e) Local view of the ② component marked in c). The scale bar in a) and c) is 1 cm.

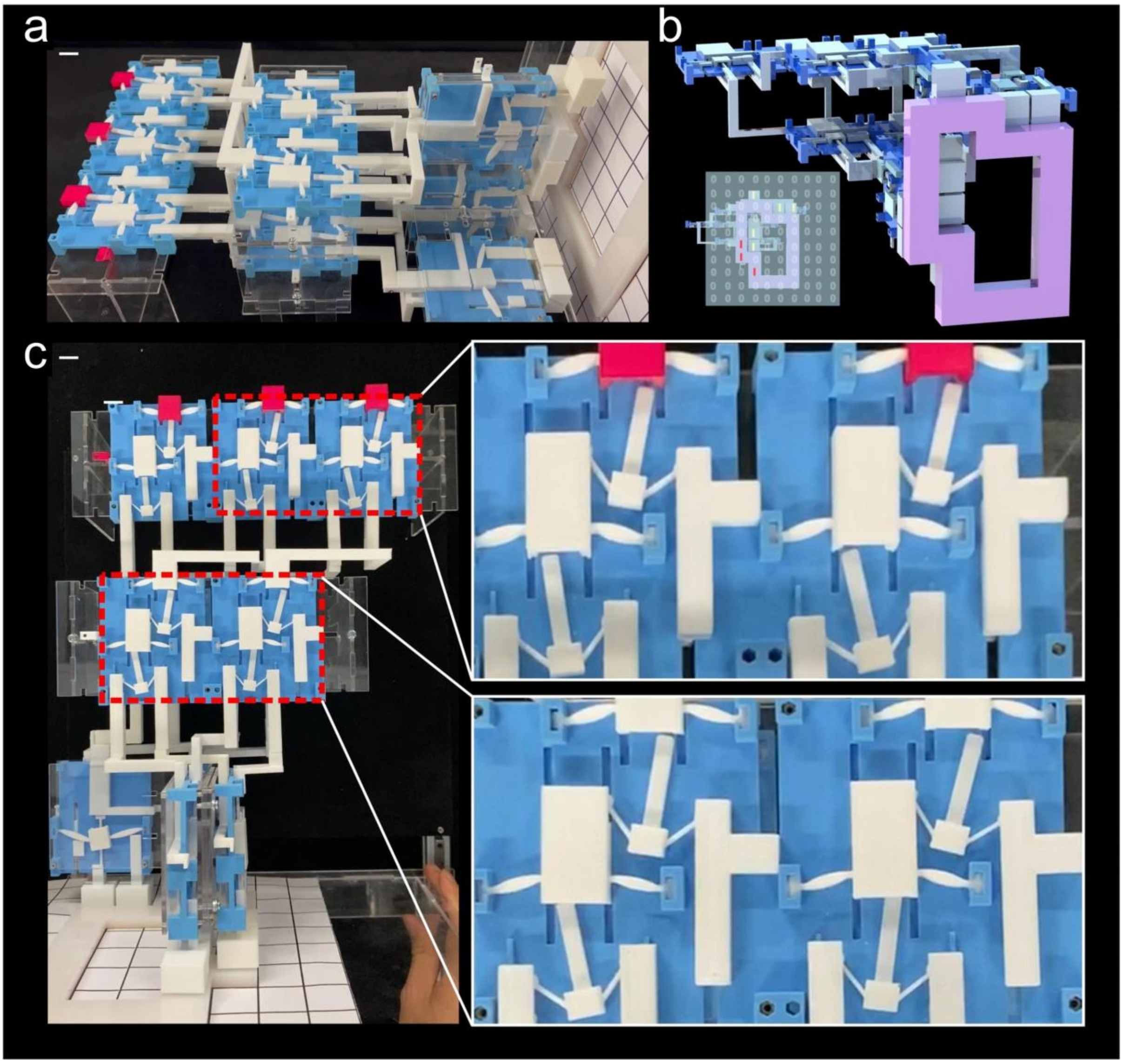

Figure S9. Functionality of the mechanical metamaterial to identify another handwritten "0". a) Photograph of the fabricated mechanical metamaterial system and its functional process to identify a 3D printed handwritten "0". b) The weight matrix pixels that were actuated by the handwritten "0". c) A front view of the mechanical metamaterial system and some local views. The scale bar in a) and c) is 1 cm.

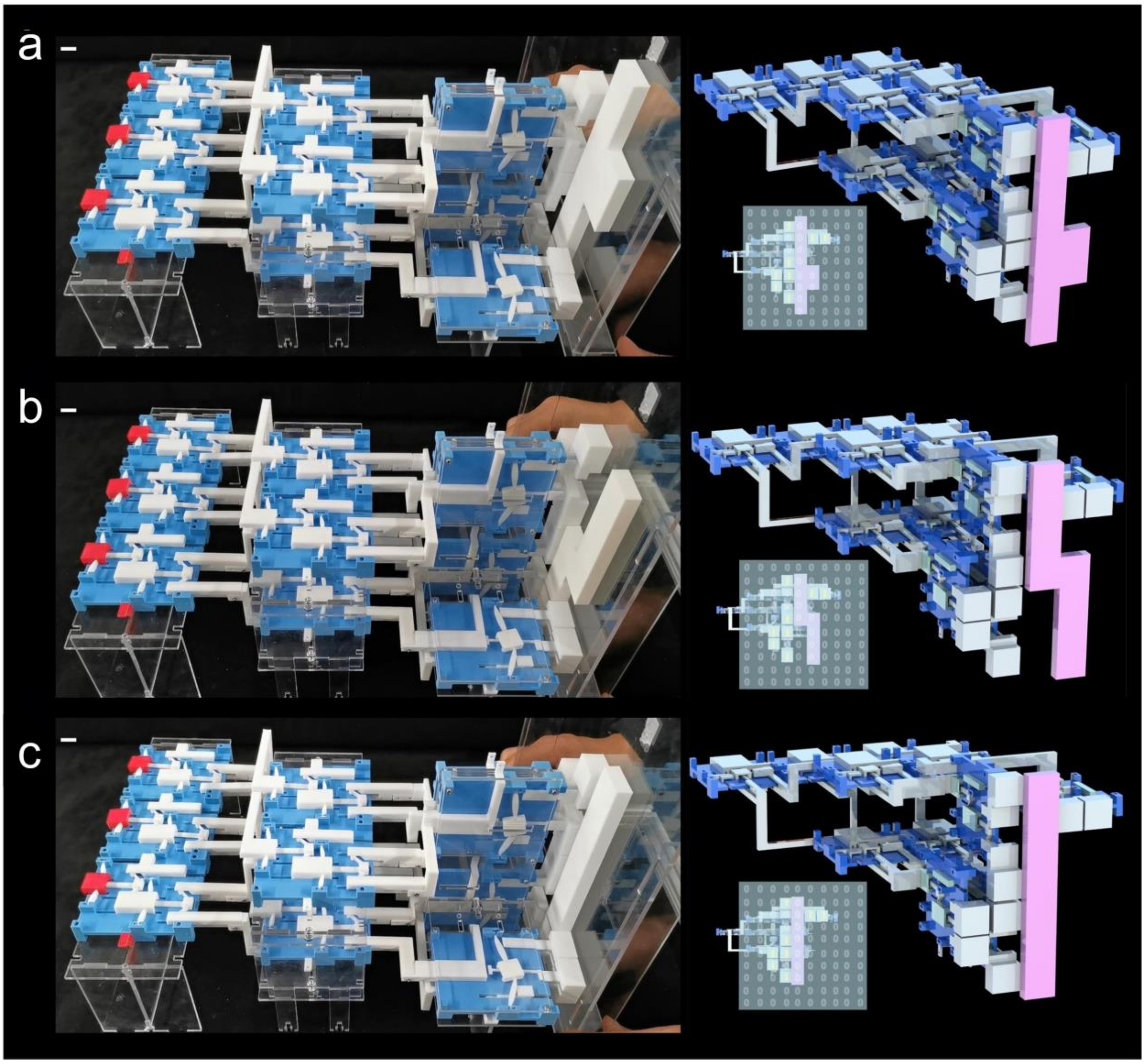

Figure S10. Functionality of the mechanical metamaterial to identify handwritten “1”. The scale bar is 1 cm.

Video S1 (separate file). Functional process of logic gates and computation modules.

Video S2 (separate file). Functional process of a full adder and full-adder computation array.

Video S3 (separate file). Reset of a full adder.

Video S4 (separate file). Functional process of the metamaterial to identify 10 handwritten “0”s.

Video S5 (separate file). Functional process of the metamaterial to identify 3 representative handwritten “1”s.

Video S6 (separate file). Testing the function of a full adder under low temperature.
